# Arm movement speed assessment via a Kinect camera: A preliminary study in healthy subjects

**DOI:** 10.1186/1475-925X-13-88

**Published:** 2014-06-27

**Authors:** Mohamed Elgendi, Flavien Picon, Nadia Magnenat-Thalmann, Derek Abbott

**Affiliations:** 1 Department of Computing Science, University of Alberta, 2-32 Athabasca Hall, T6G 2E1 Edmonton, Canada; 2 Institute of Media Innovation, Nanyang Technological University, 50 Nanyang Drive, 637553 Singapore, Singapore; 3 School of Electrical and Electronic Engineering, University of Adelaide, SA 5005 Adelaide, Australia

**Keywords:** Hand speed analysis, Kinect, Physiological movement

## Abstract

**Background:**

Many clinical studies have shown that the arm movement of patients with neurological injury is often slow. In this paper, the speed of arm movements in healthy subjects is evaluated in order to validate the efficacy of using a Kinect camera for automated analysis. The consideration of arm movement appears trivial at first glance, but in reality it is a very complex neural and biomechanical process that can potentially be used for detecting neurological disorders.

**Methods:**

We recorded hand movements using a Kinect camera from 27 healthy subjects (21 males) with a mean age of 29 years undergoing three different arbitrary arm movement speeds: fast, medium, and slow.

**Results:**

Our developed algorithm is able to classify the three arbitrary speed classes with an overall error of 5.43% for interclass speed classification and 0.49% for intraclass classification.

**Conclusions:**

This is the first step toward laying the foundation for future studies that investigate abnormality in arm movement via use of a Kinect camera.

## Introduction

Slowness in arm movement is common in many disorders, such as Huntington’s chorea [1], Parkinson’s disease [2], and cerebellar diseases [3]. However, the abnormality in arm movement varies from one disease to another. Given the vast array of disorders associated with abnormal movements, the challenge for the rehabilitation community is in obtaining high-quality evaluations at low cost.

Kinect cameras offer extremely inexpensive accurate information for sensitive motion tracking [4]. Moreover, the Kinect camera is also considered as a promising tool for the investigation of tremor and slowness in arm movements [5]. To our knowledge, there are no studies that investigate the speed of arm movement joints for detecting abnormality in arm movements using the Kinect camera or any other depth camera. However, several arm movement recognition systems have considered speed as a feature. Min et al. [6] confirmed that arm movement recognition is usually dependent on the trajectory of arm movements, and that position, speed, and curvature are useful features. Campbell et al. [7] investigated 10 different features for arm movement recognition using a Hidden Markov Model. They indicated that speed features are superior to positional features. Yoon et al. [8] used hand speed as an important feature for arm movement recognition.

Other researchers estimated the speed of arm movement using an accelerometer, for example, Rehm et al. [9] used the power of the accelerometer as a feature to classify arm movements into low- and high- speed regimes, not for diagnostic purposes. However, a recent clinical study by Howard et al. [10] explored spatial and temporal changes in shoulder motion in both asymptomatic healthy adults and rotator cuff patients with different speeds of movement by using a scapular tracking device. In contrast to those studies, in this paper we systematically explore the speed of arm movement joints with the aim of improving the classification of arm movement speed in healthy subjects. Our study builds upon Rehm’s and Howard’s work by providing a device-free analysis of arm movement, exploring the impact of different joints on the overall arm movement, and validating the system in a noisy environment.

## Materials and methods

### Ethics statement

No film recordings of subjects were made in this study. The Kinect camera provided numerical data that directly related to hand movements. Only de-identified numerical data; representing motion vectors, were stored in the database. Volunteers were researchers at the office of the Institute for Media Innovation, Nanyang Technological University, Singapore. All data are available at http://www3.ntu.edu.sg/imi/piconflavien/autres/data-speed-arm.zip and http://www.elgendi.net/databases.htm.

### Data collection

**Figure 1:**
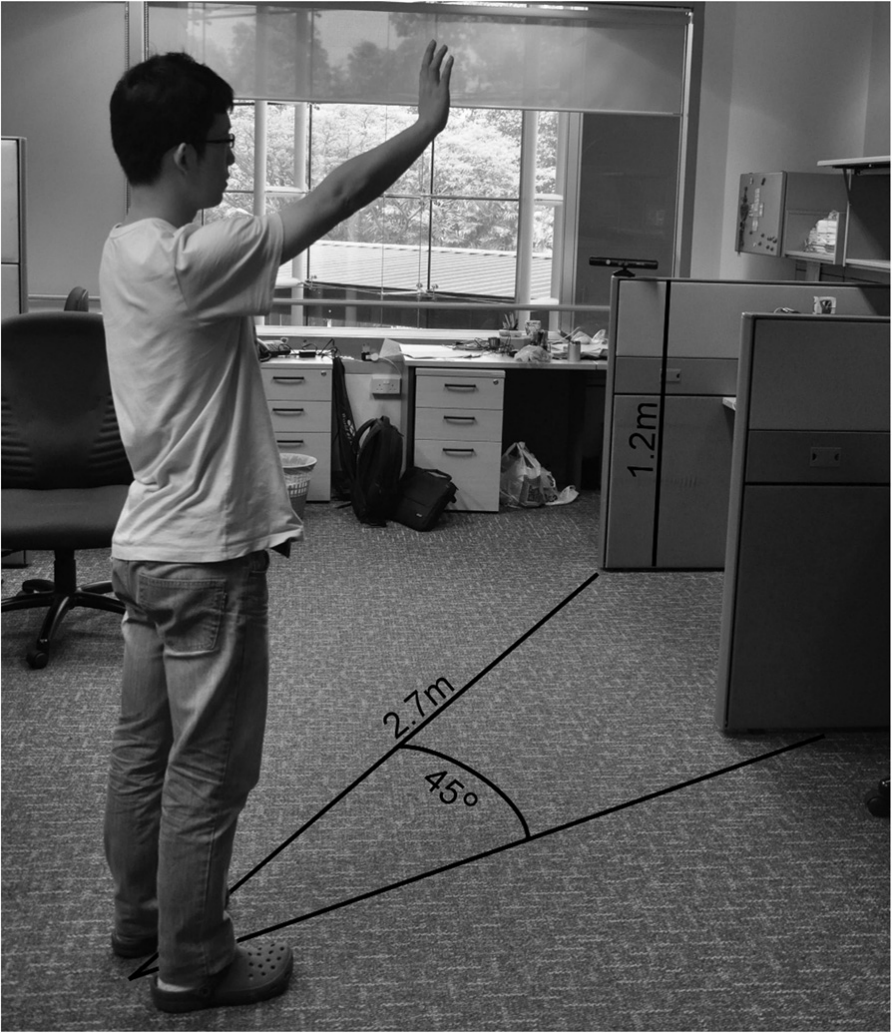
**Experimental Setup: the user faced the camera at an angle of 45° to the right of the sensor.** Every arm movement was recorded at a fixed 2.7 m distance from the camera where the Kinect camera was placed at a height of 1.2 m above the floor.

**Figure 2:**
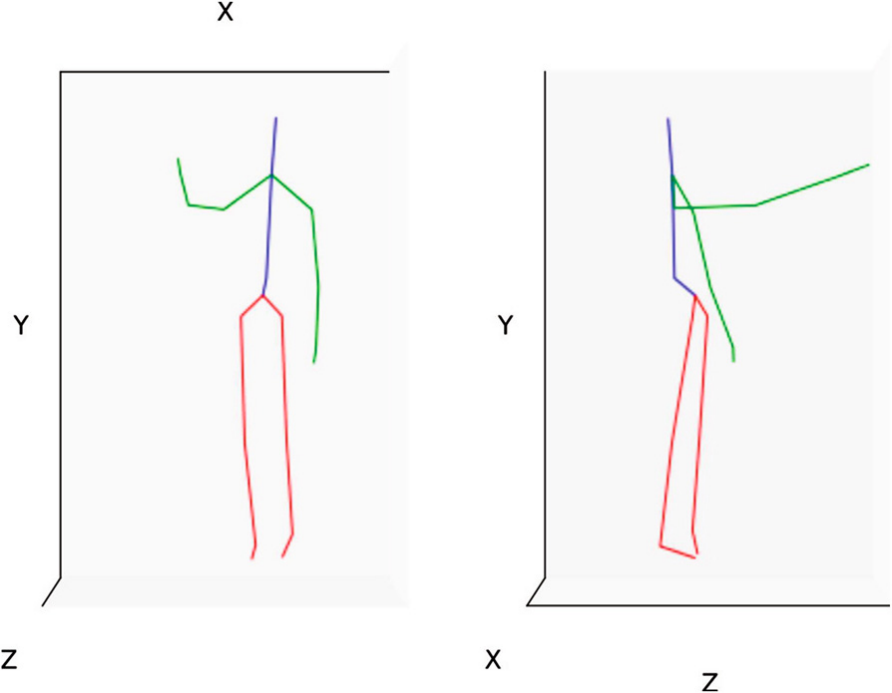
**Front and lateral view of a subject computed from the Kinect sensor data. ** This plot represents the middle of the motion and was traced using Python 2.7 and the plotting module Matplotlib 1.1.0 [12]. The instantaneous velocity is calculated using the *x*,*y*, and *z* coordinates shown in the figure. The green lines represent arms, red represents legs, and blue represents the torso.

There are currently no standard Kinect databases for arm movement analysis available to evaluate our developed algorithm. However, the Institute of Media Innovation at Nanyang Technological University has one database that contains arm movement data of 27 healthy volunteers (6 females and 21 males) with a mean ± standard deviation (SD) age of 29.7 ± 4.1 years, height of 172.9 cm ± 9.3 cm, and arm length of 71.3 cm ± 5.2 cm. Two of these volunteers were left-handed. The motion vectors were measured using a Kinect camera located 2.7 m away from the subject at a height of 1.2 m above the floor, cf. Figure 1. All Kinect data are acquired using Microsoft Kinect SDK Beta 1 (Microsoft, 2012) at a sampling frequency of 30 Hz. The Kinect device consists of a laser light source, color camera, and an infrared camera. The infrared laser source and the infrared video camera form the depth camera function, while the color video camera provides color data to the depth map. The technology was developed by PrimeSense (Tel-Aviv, Israel) and is disclosed in detail in their patents [11].During the experiment, the body of the subject faced the sensor with an angle of 45° to the right of the Kinect sensor (as seen in Figure 1). The reason behind the 45° angle is to prevent the arm joints from intersecting with the body joints, as shown in Figure 2. This generates reliable arm motion in order to study the impact of each joint of the arm on the overall speed of the right arm movement more precisely. These collected arm movements were used as a benchmark for effective speed detection of an arm movement. Measurements were taken with each subject standing vertically, with an initial position where both arms were extended along the sides of the body. Then, the subject was asked to raise his/her dominant arm. Each subject performed three sets of trials: ‘slow’, ‘normal’, and ‘fast’; with five arm movements for each set. Therefore, the number of recorded movements was 405 (27 subjects × 5 movements × 3 speeds).

For the slow movement, the subject was instructed to raise an arm as if a heavy weight was being lifted. While the three speed classes in this study were largely arbitrary, the aim was merely to demonstrate that we can achieve automatic classification of these arbitrary classes via the Kinect system. If arbitrary speed classes can be successfully distinguished, then future studies that classify the motion of healthy subjects (fast) versus those with a disorder, such as bradykinesia (slow), can be plausibly carried out using the Kinect system. Capturing the arm movement was carried out manually. In other words, the subjects waited for a signal from the recording person to start their movement and then they maintained their arm in the up position until they received a signal to return their arm to the initial position. Each recording was played back, checked, and annotated as being in one of three classes ‘slow’, ‘normal’, or ‘fast’. Two independent annotators decided the speed category of each recorded movement; when two annotators disagreed, the result was discarded and the subject was asked to repeat the experiment. The annotations were stored in a file to be compared automatically later with the speed features that will be discussed in the next section.

### Methodology

**Figure 3:**
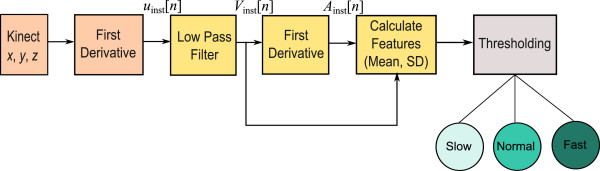
**Flowchart for the arm movement type classification.** This is the proposed algorithm that consists of three main stages: pre-processing (importing Kinect signals and first derivative), feature extraction (low-pass filter, first derivative, and calculating features), and classification (thresholding).

The proposed arm movement classification type algorithm consists of three main stages: pre-processing (resultant of coordinates as instantaneous velocity and low-pass filtering), feature extraction (calculating the first and second derivative and their mean and SD), and classification (thresholding). The structure of the algorithm is shown in Figure 3.

#### ***Pre-processing***

The Kinect body tracking software API provides the real-time position of the body joints of each user [13]. Even though we focused mainly on the skeletal joints of the arm, we chose to record the positions of all skeletal joints—center of gravity or leg movements are also potential speed indicators. With 20 joints and 3 floating point values (real numbers) representing the *x*,*y*, and *z* positions for each joint, each motion frame was expressed as a 60-element vector. The recorded joints covered all parts of the body, but we focused mainly on the arm joints: shoulder, elbow, wrist, and hand. Since the features only rely on the dynamics of the motion, there are no differences in processing data from the left or right arm. Therefore, we processed data from the joints of each subject’s dominant arm (25 right-handed and 2 left-handed).

The three-dimensional (3D) position (*x*,*y*,*z*) of a joint is expressed in the coordinate system of the Kinect camera and the units are in meters [14]. Again, the selected features rely on motion dynamics so our system is view-independent, i.e., we do not have to express the positions in the coordinate system of the subject’s body. The dynamic of each joint is computed using the variation of position of the joint over time. In the first step, each joint motion, each joint motion sequence of 3D positions, is replaced by the distance between each frame as in Eq. 1. In Figure 2, the *x*,*y*, and *z* coordinates are the position vectors of a particular joint that varies from 0 to *n*, where *n* is the number of frames in a performed motion. The instantaneous velocity of motion for a particular joint is calculated as the resultant of *x*,*y*, and *z* positions over all frames that represent a motion. The instantaneous velocity (*u*_inst_) for a given 3D motion is computed as follows:

(1) $$\begin{array}{lll} u_{\text{inst}}[\;n] & ={\left(\frac{\text{dx},y,z}{\text{dt}}\right|}_{t=\text{nT}}\; & \\ & =\frac{1}{T}\sqrt{{\left(x[\;n]-x[\;n-1]\right)}^{2}+{\left(y[\;n]-y[\;n-1]\right)}^{2}+{\left(z[\;n]-z[\;n-1]\right)}^{2}},\; \end{array}$$

where *T* is the sampling interval and equals the reciprocal of the sampling frequency and *n* is the number of motion data points.

**Figure 4:**
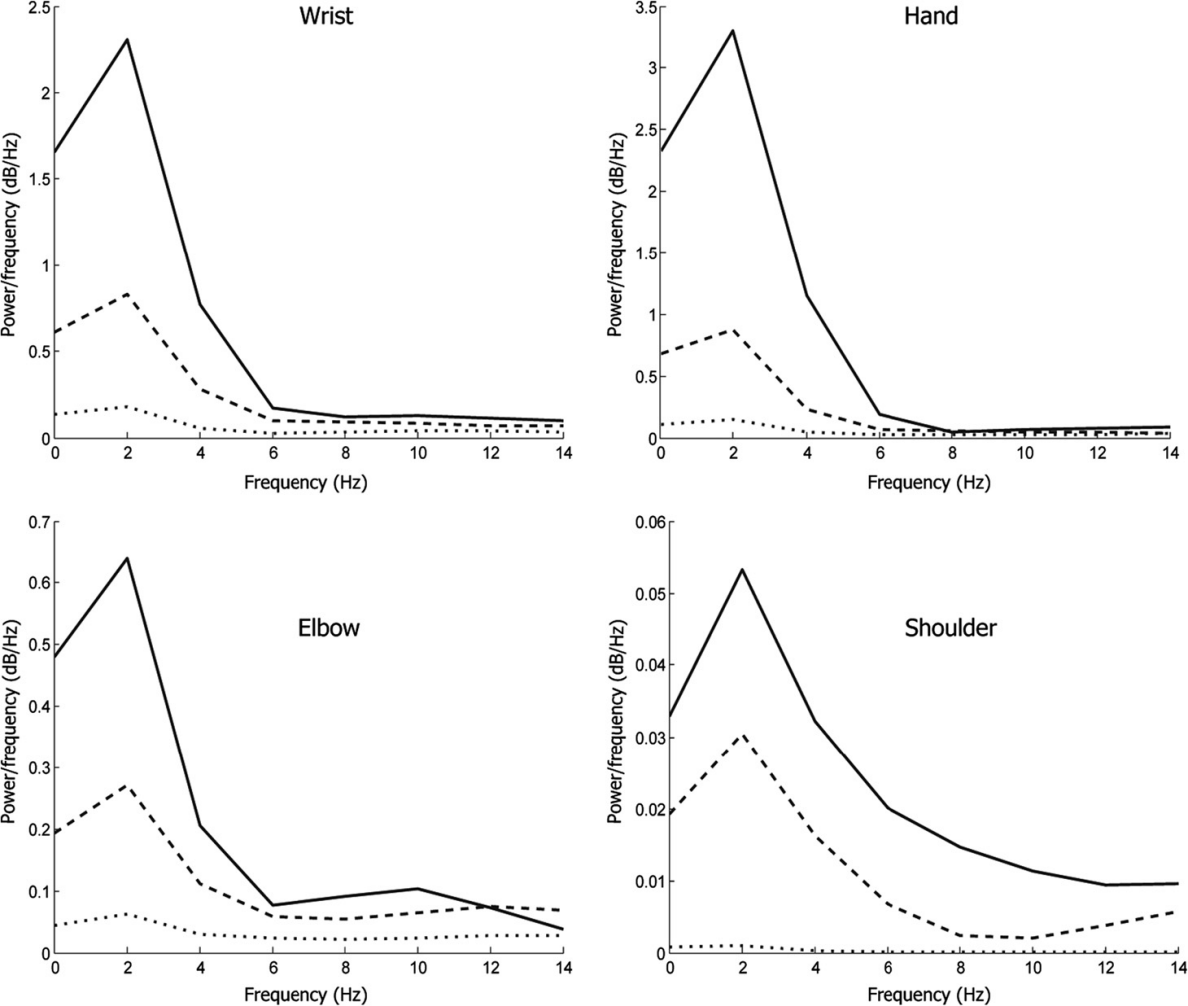
**Power spectra of the three speed motions: slow, normal, and fast.** The dotted curve represents the PSD of a slow hand movement, while the dotted curve represents the PSD of a medium hand movement. The PSD of a fast hand movement is the solid curve.

**Figure 5:**
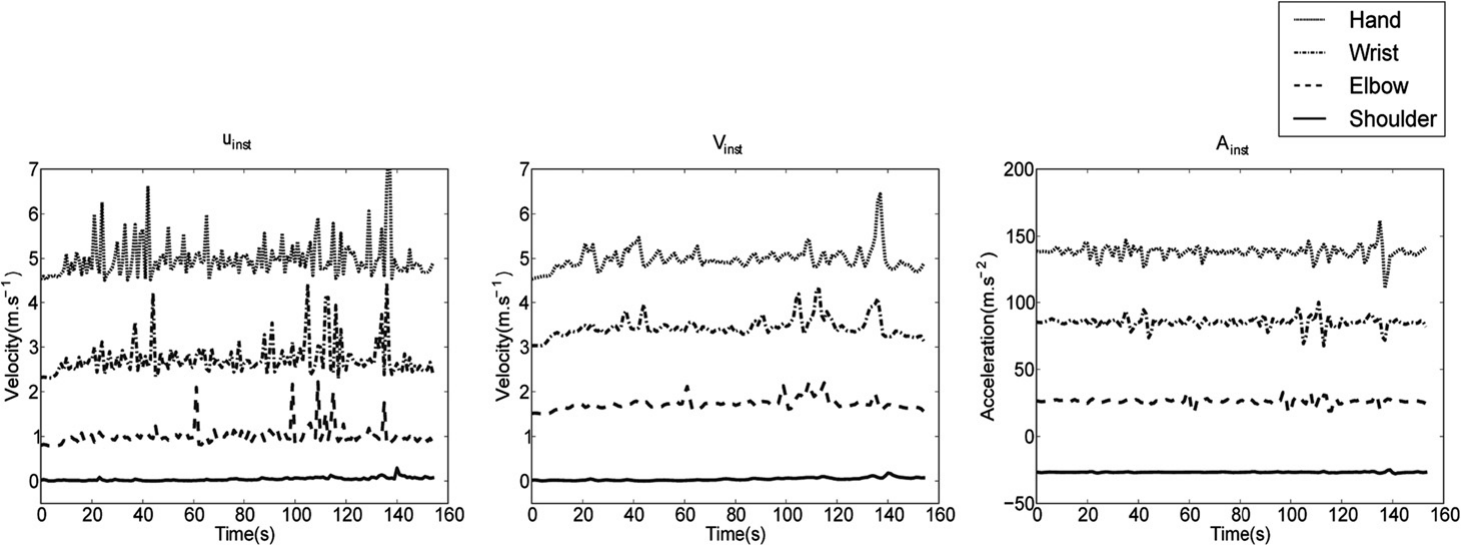
**Comparison of instantaneous non-filtered velocity (left), filtered velocity (middle), and filtered acceleration (right) of a slow motion for four joints: shoulder, elbow, wrist and hand of the right arm.** The plots are carried out for one motion of one subject. For a clearer graph, extra vertical space has been added between the plots, however the scale ratio has been preserved. From bottom to top are shoulder, elbow, wrist, and hand, respectively. The cutoff frequency of the Butterworth low-pass filter was 6 Hz.

As shown in Figure 4, the informative part of the motion lies below 6 Hz for all joints with different speed types. Thus, a low-pass filter was applied. A first-order, zero-phase bidirectional, Butterworth low-pass filter with cutoff frequency of 6 Hz was implemented. Figure 5 shows an example of the original data *u*_inst_ at the top left and the filtered data (*V*_inst_) at the top right with no phase distortion. Note that the low frequencies play a major role in identifying hand movement speed and ultimately hand tremors. The first-order filter was selected to avoid over-smoothing the acquired motion. This was carried out empirically to find a condition where the substantial part of the motion was preserved while sensor errors were strongly reduced. We decided to record the raw data, i.e., without using the pre-defined filter provided in the Kinect SDK. By doing so, we have more control over the data analysis. We then have freedom to examine the effect of filtering on the classification rate.

#### ***Feature extraction***

Before continuing the discussion of the joint signals, it is important to know which features can be extracted from a hand movement. In the literature, instantaneous velocity and acceleration have been used in diagnosing arm movements. Almeida et al. [15] examined individuals with Parkinson’s disease through the analysis of upper-limb movement at different movement frequencies and with different external timing conditions using instantaneous velocity. However, investigators [16,17] used instantaneous velocity and acceleration to investigate the movements of the finger, elbow, and shoulder during speed-aiming movements. In this paper, two features were investigated: instantaneous velocity and acceleration. The mathematical definition of instantaneous velocity (*u*_inst_) in 3D motion before filtering is described in Eq. 1, while instantaneous acceleration (*A*_inst_) is defined as:

(2) $$A_{\text{inst}}[\;n]={\left(\frac{{\text{dV}}_{\text{inst}}}{\text{dt}}\right|}_{t=\text{nT}}=\frac{1}{T}\left(V_{\text{inst}}[\;n]-V_{\text{inst}}[\;n-1]\right).$$

Although the Kinect camera is receiving increased attention, it nevertheless suffers from noise, low resolution sensors, lack of color information, and occlusion problems [18]. Therefore, it is crucial that we filter the signal to improve the classification accuracy, especially if the main goal is to determine the speed type. In our study, we computed the instantaneous velocity and instantaneous acceleration for each arm joint. Then, we calculated the following measures: average (*f*_1_,*f*_3_) and SD (*f*_2_,*f*_4_). Two features {*f*_1_,*f*_2_} are extracted from the velocity *V*_inst_ and two features {*f*_3_,*f*_4_} are extracted from the acceleration *A*_inst_. Features *f*_1_,*f*_2_,*f*_3_, and *f*_4_ are calculated as follows:

(3) $$f_{1}=(1/N)\overset{N}{\underset{n=1}{\sum }}V_{\text{inst}}[\;n],$$

(4) $$f_{2}=\sqrt{(1/N)\overset{N}{\underset{n=1}{\sum }}{\left(V_{\text{inst}}[\;n]-f_{1}\right)}^{2}},$$

(5) $$f_{3}=(1/N)\overset{N}{\underset{n=1}{\sum }}A_{\text{inst}}[\;n],$$

(6) $$f_{4}=\sqrt{(1/N)\overset{N}{\underset{n=1}{\sum }}{\left(A_{\text{inst}}[\;n]-f_{3}\right)}^{2}},$$

where *N* refers to the total number of samples in the processed motion.Figure 5 demonstrates the signal shape of four different joints of an arm movement based on the instantaneous velocity and acceleration. This is particularly interesting as it confirms that joints of the same limb have the same dynamics, especially for the hand and wrist signals. As the variance of the hand and wrist joint signals are somewhat higher compared to the elbow and shoulder signals, it is expected that the hand or the wrist signal will potentially attain higher accuracy in the classification of arm movements.

#### ***Classification***

In this section, we checked the linear separability of the calculated feature set *f *= {*f*_1_,*f*_2_,*f*_3_,*f*_4_} in both filtered and non-filtered signals. The classification steps are described in the following paragraphs.

**Figure 6:**
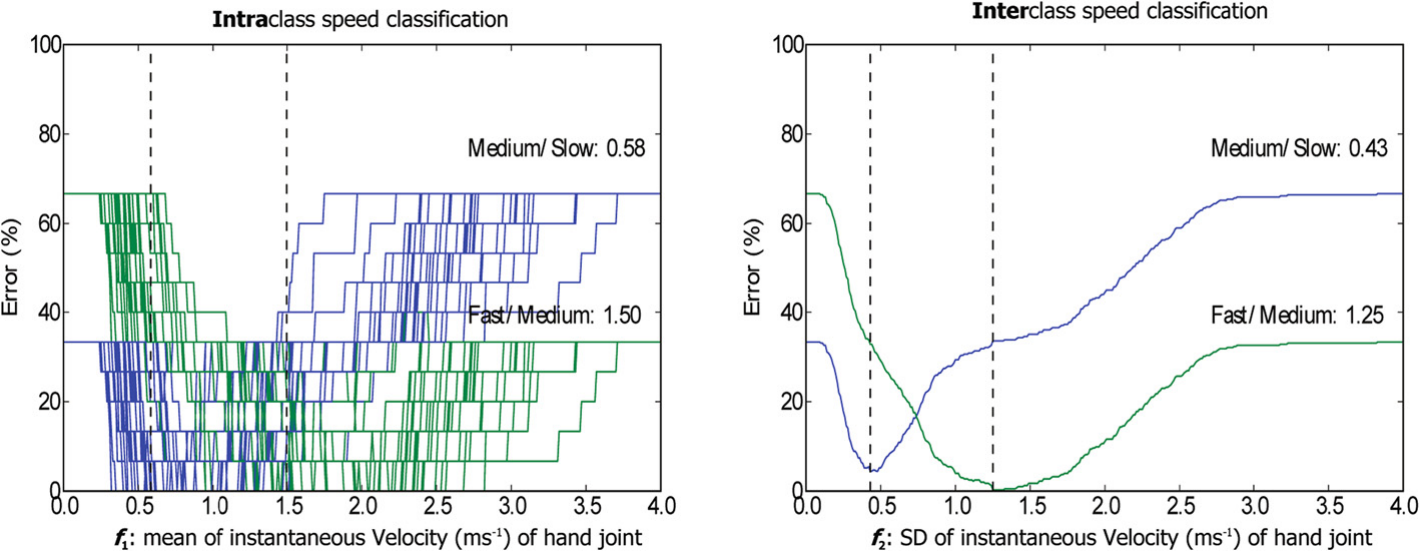
**Threshold of classification.** The figures represent, left to right, the intraclass speed classification for the mean (*f*_1_) and the interclass speed classification for the SD (*f*_2_) of the hand instantaneous velocity. The intraclass figure was made by superimposing the figure from each subject and computing thresholds using the average values of each subject threshold. The two dashed lines point to the two valleys in the figure, and their x-axis values are the used thresholds.

For each subject used as a test dataset, the thresholds and the error were reported, cf. Table 1. In this table only the results from the most relevant features were selected: mean and SD of the instantaneous velocity of the hand {*f*_1_,*f*_2_}. The results in the table show that the thresholds are quite similar for the different training datasets. There are also some subjects for whom the classification error is fairly high. These errors come from the strict separation provided by the thresholds. The first classifier is fast/medium against slow (THR_1_), while the second classifier is fast against medium/slow (THR_2_).Figure 6 demonstrates the threshold determination for inter- and intraclass speed classification. The two valleys reflect the thresholds that will be used for training the automatic speed detection. For example, for the intraclass speed classification, the slow-medium threshold was 0.58 while the medium-fast threshold was 1.50 in the non-filtered condition as shown in Figure 6 (left).

**Table 1:** Leave-one-out (LOO) cross-validation results for the best feature for intra- and interclass for the hand joint speed analysis

	**Intraclass**			**Interclass**		
	**Non-Filtered** ***f***_**1**_			**Filtered** ***f***_**2**_		
**LOO step**	**THR**_**1**_	**THR**_**2**_	**Error (%)**	**THR**_**1**_	**THR**_**2**_	**Error (%)**
1	0.57	1.47	53.33	0.43	1.25	6.66
2	0.57	1.48	60.00	0.43	1.25	0.00
3	0.56	1.48	53.33	0.43	1.25	6.66
4	0.58	1.47	26.66	0.43	1.25	0.00
5	0.59	1.52	20.00	0.47	1.25	40.00
6	0.58	1.51	13.33	0.43	1.25	0.00
7	0.59	1.51	0.00	0.48	1.25	13.33
8	0.59	1.51	0.00	0.43	1.25	20.00
9	0.58	1.50	6.66	0.43	1.25	0.00
10	0.58	1.49	13.33	0.43	1.25	0.00
11	0.58	1.49	6.66	0.47	1.25	13.33
12	0.57	1.50	13.33	0.43	1.25	13.33
13	0.58	1.48	13.33	0.43	1.25	6.66
14	0.57	1.48	66.66	0.43	1.25	0.00
15	0.58	1.47	20.00	0.43	1.25	0.00
16	0.58	1.49	26.66	0.43	1.25	6.66
17	0.58	1.50	6.66	0.43	1.25	0.00
18	0.58	1.50	13.33	0.43	1.25	6.66
19	0.59	1.50	0.00	0.43	1.25	6.66
20	0.58	1.50	0.00	0.43	1.25	0.00
21	0.58	1.49	6.66	0.43	1.25	0.00
22	0.58	1.50	0.00	0.43	1.25	0.00
23	0.58	1.49	0.00	0.43	1.25	6.66
24	0.57	1.50	33.33	0.43	1.25	6.66
25	0.59	1.50	0.00	0.43	1.25	0.00
26	0.58	1.48	20.00	0.43	1.25	0.00
27	0.57	1.49	53.33	0.43	1.25	6.66

The best feature for intra- and interclass analysis was selected based on the overall joints analysis shown in Table 1. Each LOO step means one subject is excluded from testing and the training was carried out over the remaining 26 subjects. The results of testing are displayed in the error column.

## Results and discussion

Advances in microelectromechanical systems allow measurement of the changes in velocity, position, and acceleration by enabling low-cost sensors, accelerometers, and gyroscopes. These sensors have been employed to analyze arm movement disorders.

In the literature, essential tremor typically has a frequency of 4–8 Hz when it is assessed by microelectromechanical systems, such as accelerometers [19]; however, our analysis shows frequencies that lie below 6 Hz are more informative for arm-speed assessment using using a Kinect camera.

The statistical Kruskal-Wallis and analysis of variance (ANOVA) tests allow us to investigate whether the hand-movement speed feature takes different values among the three speed classes. In the case of interclass analysis for non-filtered/filtered *f*_1_, *p *< 0.00001 was scored by the ANOVA test, while the the Kruskal-Wallis test showed significance with *p *< 0.01. On the other hand, for the intraclass analysis for filtered *f*_2_, *p *< 0.000001 was scored by the ANOVA test, while the Kruskal-Wallis test showed significance with *p *< 0.01.

In the case of interclass speed analysis, low *p*-values (*p *< 0.00000001) were scored for both tests, which indicate a large difference in the means and medians of the three speed classes. Both tests found that the three hand-movement speeds are significantly different in the case of filtered and non-filtered features. The very small *p*-value indicates that differences between the three speed classes are highly significant.

In Table 2, as expected, the hand joint was successfully classified into different speed types with the lowest error rate (0.49% for intra-classification and 5.43% for inter-classification). This result confirms the observation, shown in Figure 5, that the mean of the instantaneous velocity for the hand motion contains more information compared to the other three joints in both cases of filtered and un-filtered data. As can be seen, the hand joint is the most reliable for detecting speed in an arm movement. It is interesting to note that features based on the SD perform better than those based on the mean. Interestingly, the results of filtered and non-filtered hand-joint signals are relatively close. However, the filtered hand-joint signal scored a slightly lower classification error compared to the non-filtered signal.

**Table 2:** Error rates for non-filtered and filtered arm movement signals

		**Hand**				**Wrist**			
		***f***_**1**_	***f***_**2**_	***f***_**3**_	***f***_**4**_	***f***_**1**_	***f***_**2**_	***f***_**3**_	***f***_**4**_
		**error(%)**	**error(%)**	**error(%)**	**error(%)**	**error(%)**	**error(%)**	**error(%)**	**error(%)**
**Intra**	**Non-filtered**	**0.49**	0.98	58.27	24.69	1.48	4.44	55.80	31.85
	**Filtered**	**0.49**	2.46	58.27	11.35	1.48	4.44	55.80	15.55
**Inter**	**Non-filtered**	8.39	6.41	60.74	30.86	10.37	12.34	58.02	41.97
	**Filtered**	8.39	**5.43**	60.74	22.71	10.86	9.38	58.02	26.17
		**Elbow**				**Shoulder**			
		***f***_**1**_	***f***_**2**_	***f***_**3**_	***f***_**4**_	***f***_**1**_	***f***_**2**_	***f***_**3**_	***f***_**4**_
		**error(%)**	**error(%)**	**error(%)**	**error(%)**	**error(%)**	**error(%)**	**error(%)**	**error(%)**
**Intra**	**Non-filtered**	1.48	6.41	53.82	21.72	4.44	11.60	36.04	12.83
	**Filtered**	1.48	5.18	53.82	11.85	4.44	11.85	36.04	15.30
**Inter**	**Non-filtered**	11.35	17.53	51.60	34.32	27.40	31.35	38.51	33.58
	**Filtered**	11.35	14.56	51.60	27.90	27.40	31.11	38.51	32.09

Classification error was calculated within (intra) and between (inter) speed classes. For the intraclass speed classification, the lowest error wass achieved by either non-filtered or filtered of feature *f*_1_ (error of 0.49%), while the lowest error achieved for the interclass speed classification was 5.43% by filtered feature *f*_1_. Feature filtration is done using a Butterworth low-pass filter with a cutoff frequency of 6 Hz.

**Figure 7:**
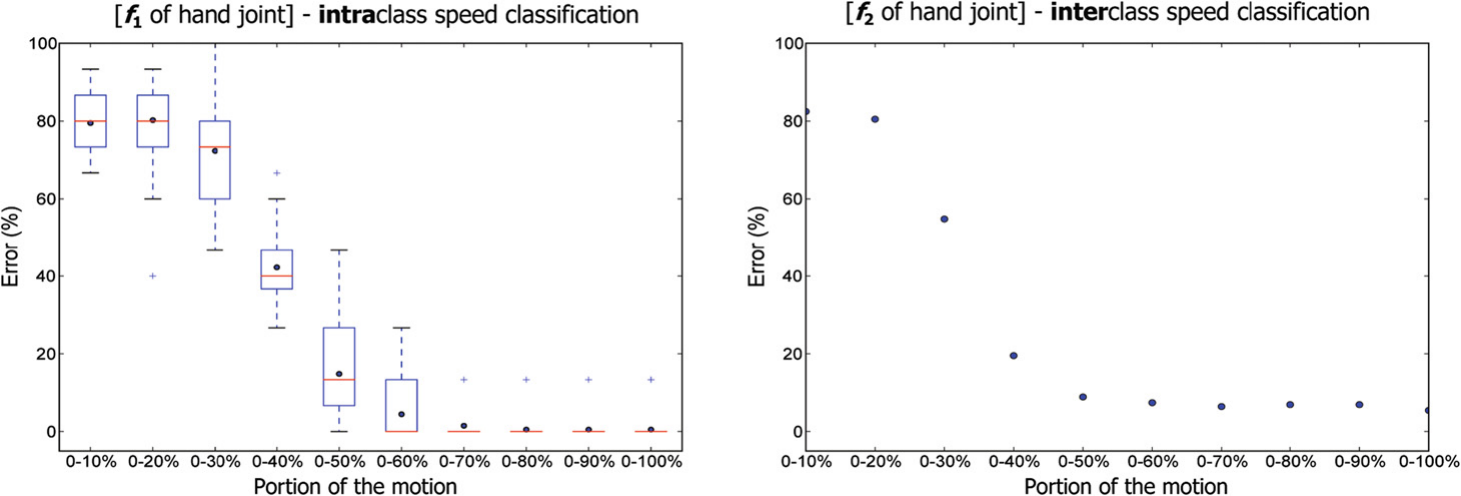
**Classification error rate of the speed types based on the percentage of the whole motion used.** This analysis was carried out to search for the most useful percentage of the whole motion that distinguishes the speed types. Knowing this percentage can lead to an effective prediction. The two figures represent, from left to right, the intraclass speed classification for *f*_1_ (mean of the instantaneous velocity) and the interclass speed classification for *f*_2_ (standard deviation of the instantaneous velocity) of the hand joint. The intraclass boxplot shows the variation within subjects. The interclass scatter shows the exact error rate over all subjects. The portion 0–50% presents a comparable error rate to the whole motion.

Is it possible to predict the speed type before the completion of a full hand movement? To answer this, we investigated the percentage of the hand movement from the start of the motion that contributes the most to the classification error. The results of this investigation are shown in Figure 7. For the inter-classification, the first 50% of the *f*_2_ feature provided an 8.8% classification error. While in the intraclass speed classification, the error rate scored by the first 60% was 4.4% using the *f*_1_ feature. This is an interesting observation as the first 50% of a motion provides low classification error and is relatively close in terms of performance to the whole motion. Knowing this fact can lead to an effective prediction, which can be carried out in real time without waiting for the whole motion to be completed. What portion of a hand-movement signal contributes the most to the classification error? Which 10% portion of the motion’s signal contains the most useful information to distinguish the speed types?

**Figure 8:**
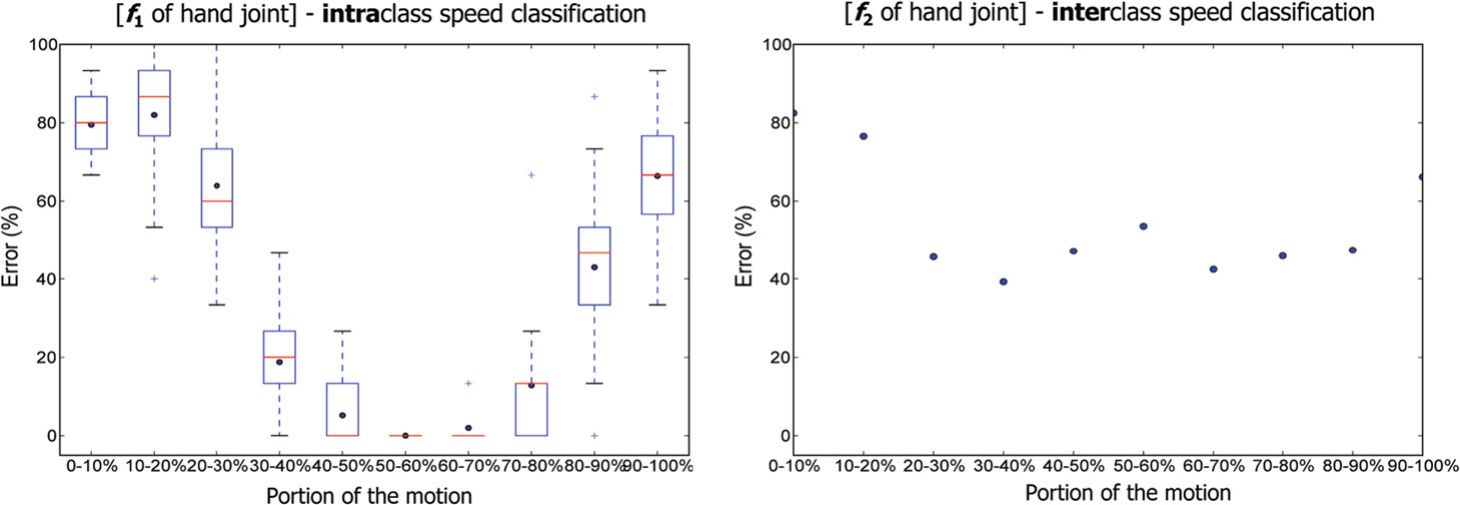
**Classification error rate of the speed types based on a sequential 10% cut of the whole motion.** This analysis was carried out to search for the most useful 10% portion of the motion’s signal that distinguishes the speed types. Knowing this percentage can lead to an effective prediction. The two figures represent, from left to right, the intraclass speed classification for *f*_1_ (mean of the instantaneous velocity) and the interclass speed classification for *f*_2_ (standard deviation of the instantaneous velocity) of the hand joint. The intraclass boxplot shows the variation within subjects, while the interclass scatter shows the exact error rate over all subjects. First, we can observe that the classification error diminishes in the middle of the curve; this seems to indicate that the most meaningful section of the motion is at the middle. The smallest error is in the portion 50–60% for both intra- and interclass speed classification.

Figure 8 shows the error rate for a sequential 10% of the motion signals. It can be seen that in the case of interclass speed classification, the portion that is 50 – 60% of the mean of the hand instantaneous velocity provides the lowest error rate of 6.4%. This is intuitive as the beginning of motion is the phase where each subject reaches a certain pose. Moreover, the intraclass analysis shows that a 0% error rate can be achieved if the 50 – 60% portion of the mean of the instantaneous velocity is used instead of the whole hand movement. This confirms that the main characteristics of a movement are determined within 50 – 60% and can be used for analysis or/and prediction. The new results offer a more direct path toward translation for clinical purposes than Bergmann et al. [20] as well as a simpler and therefore a more robust method for classification.

Studies investigating speed–amplitude relations in those with Parkinson’s disease suggest that for any given movement amplitude, the velocity is reduced [21-23]. Thus, the amplitude of the time-domain representation is used to indicate abnormal movement [24]. Interestingly, the consistent finding was the frequency power dependence of the speed of the fastest voluntary efforts; the greater the frequency power, the faster the contraction, as shown in Figure 4 in which the solid lines refer to a large frequency power associated with fast joint movements. This suggests a new indicator for abnormalities, such as bradykinesia or Parkinson’s, and the Kinect system is sensitive enough to provide classification of a joint speed.

## Limitations of study and future work

**Figure 9:**
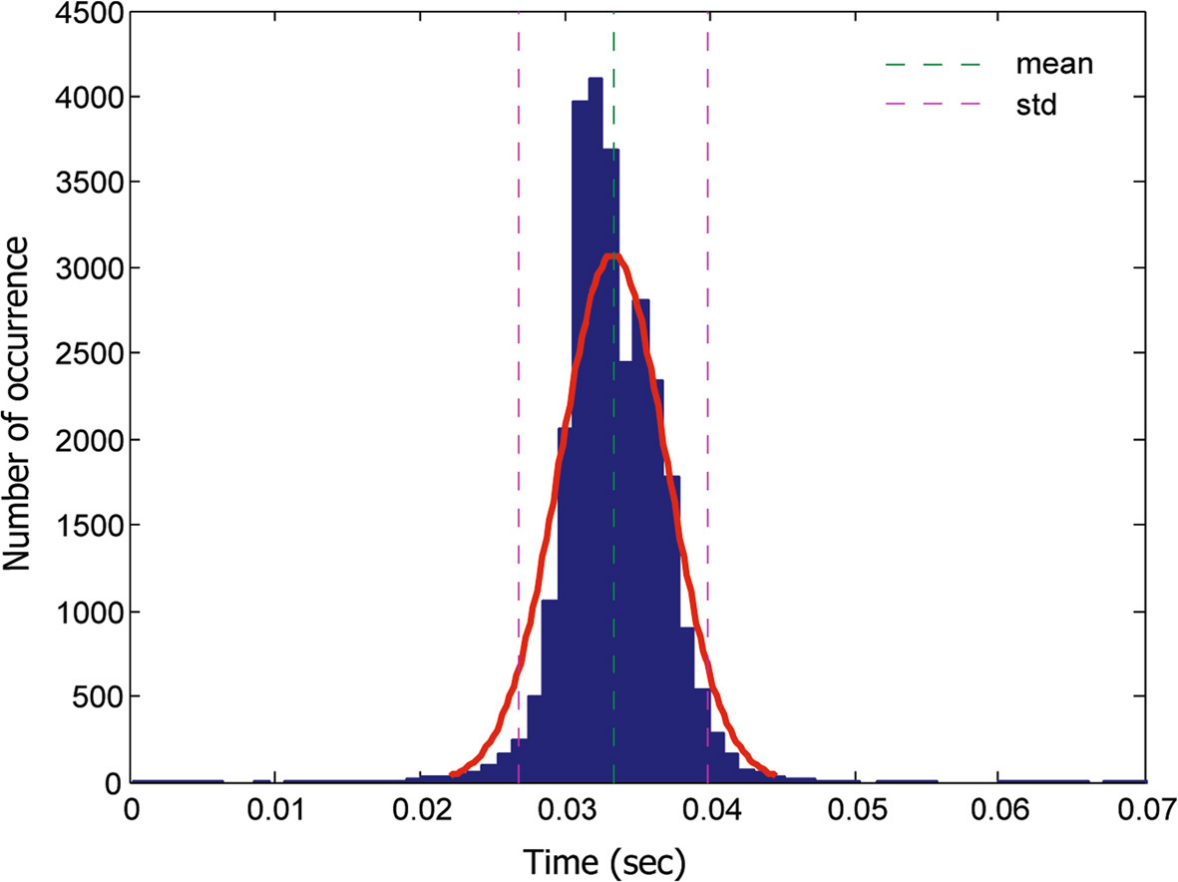
**Histogram of the timestamp difference for motion samples collected by the Kinect camera.** The histogram shows the statistical analysis of the time difference for motion samples collected by the Kinect camera. The mean sampling time was 0.0334 sec (sampling frequency of 29.94 Hz), while the sampling error is ± 0.0037 sec; this implies a sampling frequency error of 2.7 Hz. A Gaussian curve (red color) has been fitted on the data.

In this study, mimicking unhealthy motion provided initial validation of the system—this is a necessary step before assessment of real patients at a hospital/clinic. Thus, we recommend that future work examine our method on patients that suffer from hand tremor as the thresholds calculated in this study are based on healthy subjects. A larger sample size and a diverse set of tremor movements are needed in order to generalize the findings of this study. To our knowledge, there is no available Kinect database with measurements from patients with hand tremor. In future studies it may be advisable to test the optimal distance for positioning the Kinect camera as sometimes subjects cannot be detected if they are relatively close to the camera. It would also be useful to know how accurately the Kinect camera can estimate speed of an arm movement compared to the speed of an arm movement with a benchmark standard (such as a 3D analysis system, e.g., Vicon, Optitrak, etc.). Perhaps combining all the features together to build a single classifier to separate speed classes can be carried out in the future.Figure 9 shows that the sampling rate of the Kinect camera is relatively unstable; It fluctuates between 26.95 Hz and 33.67 Hz rather than sampling the data at a consistent frequency of 30 Hz. Our results show that this is not an issue unless high-frequency analysis is needed, which is not the main concern of our study. However, the use of smoothing techniques decreases the impact of sensor instability. In cases where greater sampling stability is required, future studies can consider modifying the hardware with a more stable frequency source.

We developed a real-time system that uses 50–60% of the hand movement. However, further investigations with subjects possessing abnormal hand speeds are needed. Exploring simple features, such as the mean and SD of the motion, is promising in terms of computational complexity and efficiency. However, this can be further improved by investigating other features in the time and frequency domains. Thresholds provide a strict separation between the data; the resulting misclassification can potentially be reduced by introducing an acceptance interval around the threshold values.

## Conclusion

In this paper we presented a speed analysis of arm movement. Results show that 1) instantaneous velocity provides more reliable classification compared to instantaneous acceleration, 2) the mean is a better feature compared to the standard deviation for instantaneous velocity, and 3) the hand joint is the most efficient joint for speed detection in arm motion. Moreover, a low-pass filter improves the interclass speed classification but has no effect on the intraclass classification. For interclass speed classification, the mean of non-filtered instantaneous velocities scored a 0.49% error rate in detecting the speed type over 405 motions, while the standard deviation of filtered instantaneous velocity scored 5.43% during the intraclass classification. Moreover, the first 60% of the range of movement provides a classification error relatively close to the use of the whole movement range—this can be used for predicting the speed type in real time. Furthermore, the most important 10% of a whole motion is the 50–60% region. The results are promising, and this approach can be implemented in a human–computer intraction system for interactive tremor diagnosis, specifically measuring hand-related disability and improvement. In this study, we asked healthy subjects to mimic abnormality by moving slowly; however, testing this approach on patients with Parkinson’s disease or any hand tremors remains a task for future work.

## Consent

Written informed consent was obtained from each subject for the publication of this report and any accompanying images.

## Competing interests

The authors declare that they have no competing interests.

## Authors’ contributions

ME designed the experiment. ME and FP carried out the data collection. ME, FP, DA, and NT performed the statistical analysis. ME, FP, DA, and NT conceived of the study, and participated in its design and coordination and helped to draft the manuscript. All authors read and approved the final manuscript.
